# Translated and culturally adapted internet-delivered cognitive therapy for social anxiety disorder in Japanese clinical settings: study protocol for a randomised controlled trial

**DOI:** 10.1186/s13063-024-08303-6

**Published:** 2024-07-19

**Authors:** Naoki Yoshinaga, Graham R. Thew, Yuta Hayashi, Hiroki Tanoue, Michikazu Nakai, David M. Clark

**Affiliations:** 1 School of Nursing, Faculty of Medicine, University of Miyazaki, 5200, Kihara, Kiyotake, Miyazaki City, 889-1692 Japan; 2 Department of Experimental Psychology, University of Oxford, The Old Rectory, Paradise Square, Oxford, OX1 1TW UK; 3 Oxford Health NHS Foundation Trust, Oxford, UK; 4 Department of Nursing, Graduate School of Health Sciences, Kobe University, 7-10-2 Tomogaoka, Suma-Ku, Kobe, Hyogo 654-0142 Japan; 5 Division of Data Management, Department of Social Medicine, Faculty of Medicine, University of Miyazaki, 5200, Kihara, Kiyotake, Miyazaki City, 889-1692 Japan; 6 Clinical Research Support Center, Faculty of Medicine, University of Miyazaki Hospital, University of Miyazaki, 5200, Kihara, Kiyotake, Miyazaki City, 889-1692 Japan

**Keywords:** Clinical trial protocol, Cognitive behavioural therapy, Cognitive therapy, Cultural adaptation, Internet interventions, Japan, Randomised controlled trial, Social anxiety disorder

## Abstract

**Background:**

Cognitive therapy for social anxiety disorder (CT-SAD) has extensive empirical support and is recommended in several national guidelines. However, ensuring access to evidence-based psychological therapies such as CT-SAD remains a global challenge. An internet-delivered version of this treatment protocol (iCT-SAD) has recently been developed in the UK as a way to overcome this challenge, demonstrating comparable outcomes to face-to-face CT-SAD whilst requiring less therapist time per client. Initial findings also suggest its cross-cultural transferability, but the previous studies in other cultural settings used the English language programme and only included English-fluent participants as a second language. It is not yet known what outcomes can be achieved once the programme has been translated and adapted for a different cultural context. Therefore, this trial aims to evaluate the clinical efficacy of Japanese iCT-SAD when combined with treatment as usual (TAU) in clients with SAD.

**Methods:**

This two-arm, parallel-group, superiority randomised controlled trial will recruit 60 Japanese participants with SAD, randomly assigning them to either Japanese iCT-SAD + TAU or TAU alone at a ratio of 1:1. The primary outcome measure is the self-report Liebowitz Social Anxiety Scale, and secondary.outcomes include other measures of social anxiety symptoms and processes, general mood and functioning, and response to treatment. We will also assess treatment acceptability and gather participant feedback. Assessments will take place at baseline (week 0), mid-treatment (week 8), and post-treatment (week 15), with a further 3-month follow-up (week 27) for the iCT-SAD + TAU arm. The primary analyses will be conducted on an intent-to-treat basis, comparing the primary and secondary outcome measures between groups using linear mixed-effect models, along with additional mediation analysis.

**Discussion:**

Investigating the efficacy of translated and culturally adapted iCT-SAD in different cultural contexts is an important step in evaluating the global reach of internet interventions. This trial will provide valuable insights into the effects of iCT-SAD combined with usual care, and how this treatment could be delivered in routine clinical settings in Japan.

**Trial registration:**

International Standard Randomized Controlled Trials (ISRCTN), ISRCTN82859645, registered on January 19, 2024. UMIN Clinical Trials Registry (UMIN-CTR), UMIN000052702, registered on November 6, 2023.

## Administrative information

Note: the numbers in curly brackets in this protocol refer to SPIRIT checklist item numbers. The order of the items has been modified to group similar items (see http://www.equator-network.org/reporting-guidelines/spirit-2013-statement-defining-standard-protocol-items-for-clinical-trials/).

**Table:** 

Title {1}	Translated and culturally adapted internet-delivered cognitive therapy for social anxiety disorder in Japanese clinical settings: study protocol for a randomised controlled trial
Trial registration {2a and 2b}.	International Standard Randomized Controlled Trials (ISRCTN), ISRCTN82859645, registered on January 19, 2024. UMIN Clinical Trials Registry (UMIN-CTR), UMIN000052702, registered on November 6 2023.
Protocol version {3}	Protocol version 1.0, 2023-October-18
Funding {4}	This trial is supported by the Grants-in-Aid for Scientific Research from the Japan Society for the Promotion of Science (JSPS) (20H01769 to NY), the Daiwa Anglo-Japanese Foundation (13,068 and 13,890 to GRT), the Wellcome Trust [200796 to DMC], and the Oxford Health NIHR Biomedical Research Centre.
Author details {5a} * Correspondence (NY: naokiy@med.miyazaki-u.ac.jp; GRT:graham.thew@psy.ox.ac.uk)	Naoki Yoshinaga^1*^, Graham R. Thew^2,3*^, Yuta Hayashi^4^, Hiroki Tanoue^1^, Michikazu Nakai^5,6^, David M. Clark^2^ ^1^ School of Nursing, Faculty of Medicine, University of Miyazaki, 5200 Kihara, Kiyotake, Miyazaki City, Miyazaki 889–1692, Japan ^2^ Department of Experimental Psychology, University of Oxford, The Old Rectory, Paradise Square, Oxford OX1 1TW, UK ^3^ Oxford Health NHS Foundation Trust, Oxford, UK ^4^ Department of Nursing, Graduate School of Health Sciences, Kobe University, 7–10-2 Tomogaoka, Suma-ku, Kobe, Hyogo 654–0142 Japan ^5 ^Division of Data Management, Department of Social Medicine, Faculty of Medicine, University of Miyazaki, 5200 Kihara, Kiyotake, Miyazaki City, Miyazaki 889–1692, Japan ^6^ Clinical Research Support Center, University of Miyazaki Hospital, Faculty of Medicine, University of Miyazaki, 5200 Kihara, Kiyotake, Miyazaki City, Miyazaki 889–1692, Japan
Name and contact information for the trial sponsor {5b}	This investigator-initiated trial is sponsored by the Faculty of Medicine, University of Miyazaki, 5200 Kihara, Kiyotake, Miyazaki City, Miyazaki 889–1692, Japan.
Role of sponsor {5c}	The funding sources had no role in the design of this trial and will not have any role during its execution, analyses, interpretation of the data, or decision to submit results.

## Introduction

### Background and rationale {6a}

Social anxiety disorder (SAD) is a common and chronic mental health problem, which typically has an early age of onset, leading to a substantial negative impact on individuals’ quality of life [1]. Individual face-to-face cognitive therapy for SAD (CT-SAD) based on the Clark and Wells model [2] has extensive empirical support [3] and as a result is recommended in national guidelines in both the UK [4] and Japan [5]. Although there is evidence to suggest that people with mental health problems prefer psychological over pharmacological treatments [6], ensuring access to evidence-based psychological therapies such as CT-SAD remains a global challenge [7].

In the UK, an internet-delivered version of this treatment protocol (iCT-SAD) was developed to support wider access and dissemination [8, 9]. In order to increase the number of people receiving empirically supported psychotherapies, the National Institute for Health and Care Excellence (NICE) has conditionally recommended iCT-SAD for use in the national health service [10]. iCT-SAD is a therapist-guided internet intervention, which uses a modular structure to deliver the same treatment components as face-to-face CT-SAD. A randomised controlled trial in the UK found it to show strong efficacy, with outcomes comparable to face-to-face CT-SAD, but requiring much less therapist time per client [8]. Results of a pilot trial and randomised controlled trial in Hong Kong showed outcomes comparable to the UK trial, which suggests that it is possible to transport iCT-SAD between cultures without a substantial drop in efficacy [11, 12].

As the Hong Kong trials used the English version of iCT-SAD and included only English-speaking participants, it is not yet known what outcomes can be achieved once the programme has been translated and adapted for a different cultural context such as Japan. Initial evidence has suggested that the translation from English to Japanese has been achieved successfully, incorporating a number of small cultural adaptations where appropriate [13]. A subsequent single-arm pilot trial indicated that Japanese iCT-SAD showed good acceptability to clients and preliminary evidence of strong efficacy in Japanese clinical settings [14]. A randomised controlled trial is now warranted to evaluate Japanese iCT-SAD more robustly. The primary aim of this trial is to examine the clinical efficacy of the translated and culturally adapted Japanese iCT-SAD when combined with treatment as usual in Japanese individuals with SAD through a randomised controlled design.

## Objectives {7}

The primary objective of the current trial is to examine whether a Japanese version of iCT-SAD in combination with treatment as usual (iCT-SAD + TAU) is superior to TAU alone.

The secondary objectives are as follows:

- To examine whether the Japanese iCT-SAD programme is acceptable to clients
- To evaluate how the outcomes of the iCT-SAD + TAU intervention compare to previous studies of the iCT-SAD programme in the UK and Hong Kong
- An exploratory examination of whether baseline clinical and demographic characteristics are associated with clinical outcomes in the iCT-SAD + TAU arm
- An exploratory examination of candidate mediators of the relationship between treatment arm and clinical outcome

## Trial design {8}

This trial employs a two-arm, parallel-group, superiority randomised controlled design, comparing Japanese iCT-SAD in combination with treatment as usual (iCT-SAD + TAU) to TAU alone. Participants will be allocated using a 1:1 ratio. Participants will not be blinded to treatment allocation.

## Methods: participants, interventions and outcomes

### Study setting {9}

Participants will be recruited from outpatient sites at different psychiatric hospitals and clinics across Japan via referral from their primary psychiatrist. All screening and assessments will be conducted remotely or in person, and iCT-SAD will be provided on a dedicated online platform. TAU from their primary psychiatrist will be provided in person. Questionnaires for measuring outcomes and post-treatment feedback from participants will be collected online.

### Eligibility criteria {10}

The inclusion criteria for participants are as follows:

- Primary diagnosis of SAD based on the Diagnostic and Statistical Manual of Mental Disorders, Fifth Edition (DSM-5)
- Aged 18 years or over
- Regular, private access to an appropriate internet enable device
- Resident in Japan and proficient in Japanese (written and spoken)
- Able to visit their primary psychiatrist regularly during the trial (at least monthly in person) for safety monitoring
- Participant not currently undertaking other structured psychological therapy and agrees not to start such interventions during the trial

The exclusion criteria for participants are as follows:

- Current psychosis, bipolar disorder, antisocial personality disorder, or alcohol/substance use disorder (moderate or severe); this comorbidity is likely to interfere with the client’s ability to use the treatment website regularly and to process and apply the treatment content
- Active suicidal ideation with intent or plan; this would indicate that immediate support is required to ensure the client’s safety and that allocation to the intervention or control arm would not be sufficient at that time
- Previously received CT or cognitive behavioural therapy for SAD (defined as at least five sessions and including an exposure component); this may confound the study’s outcomes, especially with regard to iCT-SAD

### Who will take informed consent? {26a}

Informed consent will be obtained by the assessor during the eligibility assessment. Prior to this assessment, potential participants will have been sent the trial information sheet, which includes details of the purpose, design, criteria, and protection of personal information. During the eligibility assessment, potential participants will have the opportunity to ask questions about the trial. If they are happy to proceed, informed consent will be taken electronically by them submitting an online form. This form includes checkboxes for participants to state that they have received the trial information and agree to participate in the trial.

### Additional consent provisions for collection and use of participant data and biological specimens {26b}

The consent form includes permission to store an anonymised version of the dataset in an online repository. No biological specimens will be collected.

## Interventions

### Explanation for the choice of comparators {6b}

The comparator for this trial is TAU alone. TAU consists of standard psychiatric care, where participants visit their regular psychiatrist on a monthly basis. These appointments focus on mood and symptom monitoring, ongoing assessment of safety and clinical risk, and medication management. TAU was chosen as the comparator as it represents standard practice for the treatment of SAD in Japan. Japanese government regulations and implementation guidelines require that any internet-delivered intervention must be combined with regular face-to-face ambulatory care provided [15, 16]. TAU as described therefore represents an appropriate comparator for the intervention arm, which combines iCT-SAD with TAU. After the post-assessment, participants in the control arm (TAU alone) will be offered the option to receive iCT-SAD.

### Intervention description {11a}

The intervention for this trial is iCT-SAD combined with TAU (iCT-SAD + TAU) (see the above item 6b for more details about TAU). iCT-SAD is an internet-delivered therapist-guided intervention (see ‘Introduction’) [8, 9]. iCT-SAD contains eight core modules which cover key concepts on the nature and maintenance of social anxiety problems, plus 16 optional modules covering common beliefs and difficulties associated with SAD. Participants are supported to complete the core modules across the first two weeks of treatment. Subsequently, the therapist releases those optional modules that are particularly relevant to each participant’s concerns. Participants are also supported to conduct ‘behavioural experiments’, in which they test out their feared concerns in social situations whilst focussing their attention externally and dropping their safety behaviours. Trained therapists support participants by telephone, text messages, and video calls via webcam. Each telephone call lasts approximately 20 min and is used to review questionnaire measures, progress with modules and behavioural experiments, and planning for the coming week. To support initial engagement, two phone calls per week are scheduled during the first 2 weeks of treatment, followed by once-weekly phone calls until the end of the 14-week treatment phase. During the subsequent follow-up period, up to three booster calls are scheduled at monthly intervals.

### Criteria for discontinuing or modifying allocated interventions {11b}

If participants have a concern about their treatment or participation in the trial, they will be encouraged to discuss this with their therapist in the first instance. The therapist and trial team will then consider whether these concerns can be addressed through more careful tailoring of the intervention to the participant’s needs. If this is not possible and the participant wishes to withdraw from the trial, they are free to do so.

If negative events occur that require some form of urgent alternative treatment (e.g. substance misuse, deliberate self-harm, suicidal ideation or attempts), the project lead will be informed immediately to consider appropriate actions, liaising with the participant’s primary psychiatrist to arrange onward referrals.

### Strategies to improve adherence to interventions {11c}

All trial therapists have previous experience of using cognitive therapy approaches to treat SAD. They have received specialist training in iCT-SAD from an expert in this treatment protocol and have experience of treating clients using iCT-SAD from a recent pilot trial. All therapists will receive regular group supervision throughout the trial, which will include reviewing therapists’ use of the iCT-SAD site to support adherence to protocol. Monthly supervision-of-supervision will be provided by the UK team to ensure delivery is consistent with previous studies of iCT-SAD.

Participants are supported to adhere to the intervention protocol in a number of ways. Treatment begins with a more intensive phase in the first 2 weeks, supporting participants to make a strong start on their treatment, to develop engagement and motivation. Their therapist can review their progress working through treatment modules and behavioural experiments and will provide encouragement, help, and reminders as necessary. Therapists and supervisors will review participants’ activity on the iCT-SAD regularly. This provides an effective method for adherence monitoring, for example by examining whether modules or other therapy exercises have been completed.

### Relevant concomitant care permitted or prohibited during the trial {11d}

There are no restrictions on the TAU component of each treatment arm. Each primary psychiatrist will continue to provide usual treatment as clinically indicated, including medication changes if necessary. However, the initiation of other structured psychological therapy is prohibited in order to measure directly the effectiveness of iCT-SAD. All treatment changes, with the reasons for these changes, will be recorded throughout the trial period.

### Provisions for post-trial care {30}

Following trial participation, all participants will remain under the care of their primary psychiatrist, who will review their clinical needs and arrange further treatment as appropriate.

### Outcomes {12}

The principal assessment points for outcome measures will be baseline (week 0), mid-treatment (week 8), post-treatment (week 15), and 3-month follow-up (week 27, intervention arm only). The primary assessment timepoint is post-treatment (week 15).

#### Primary outcome measure

The primary outcome is a self-report version of the Liebowitz Social Anxiety Scale (LSAS) [17], one of the most widely used scales to assess the severity of social anxiety.

#### Secondary outcome measures

Secondary outcome measures will be as follows.

### Social anxiety symptoms and processes

The proportion of participants who no longer meet the SAD diagnostic criteria will be evaluated using the Anxiety and Related Disorders Interview Schedule for DSM-5 (ADIS-5) [18, 19] conducted by an independent assessor blinded to the treatment arm at the post-treatment time point. Psychological processes that maintain SAD in the Clark and Wells model [2] will be assessed using the Social Cognitions Questionnaire (SCQ), Social Behaviour Questionnaire (SBQ), Social Attitudes Questionnaire (SAQ), and Social Phobia Weekly Summary Scale (SPWSS), as described in Clark (2005) [20]. Social participation and social satisfaction will also be assessed [21].

### General mood and functioning

Depressive symptoms will be assessed with the Patient Health Questionnaire (PHQ-9) [22, 23], generalised anxiety symptoms with the Generalised Anxiety Disorder Questionnaire (GAD-7) [24, 25], and functional impairments with the Work and Social Adjustment Scale (WSAS) [26].

### Response to treatment

Response to treatment, remission from SAD, and reliable deterioration are defined using the criteria described in a pilot trial in Japan [14], which are based on previous studies of iCT-SAD [8, 9, 11, 12]. Response is defined as an improvement in the LSAS between pre- and post-treatment greater than 31%. This is comparable to an improvement subscore of 2 ‘much improved’ on the Clinical Global Impression, a psychiatric measure commonly used to define a response. Remission is defined as a decrease in the LSAS score of at least 12 points and post-treatment score of 38 points or less. Reliable deterioration is defined as an increase of at least 12 points in the LSAS score.

### Acceptability

To examine the acceptability of iCT-SAD, we will also evaluate the dropout rate, rates of module completion, and participants’ feedback about their experience with the intervention.

### Participant timeline {13}

Participant enrolment will proceed in three stages as described below.

### Stage 1

Psychiatrist discusses the trial with a potential participant and makes a referral to the trial team if appropriate. They provide relevant contact details, clinical assessment information, and confirmation that they are able to provide ongoing TAU throughout the trial. The trial office will then send the potential participant the trial information, eligibility screening questions, and initial questionnaire pack to complete and return. Eligible participants are then invited for an assessment session.

### Stage 2

The assessment session is conducted by one of the trial therapists in person or via videoconference. It will confirm the primary diagnosis of SAD using ADIS-5, assess comorbid conditions using the Structured Clinical Interview for DSM-5 Disorders (SCID-5) [27], and assess eligibility criteria, risk, and current medication. Informed consent will be taken electronically. The trial team will then discuss whether the participant is eligible.

### Stage 3

**Fig. 1:**
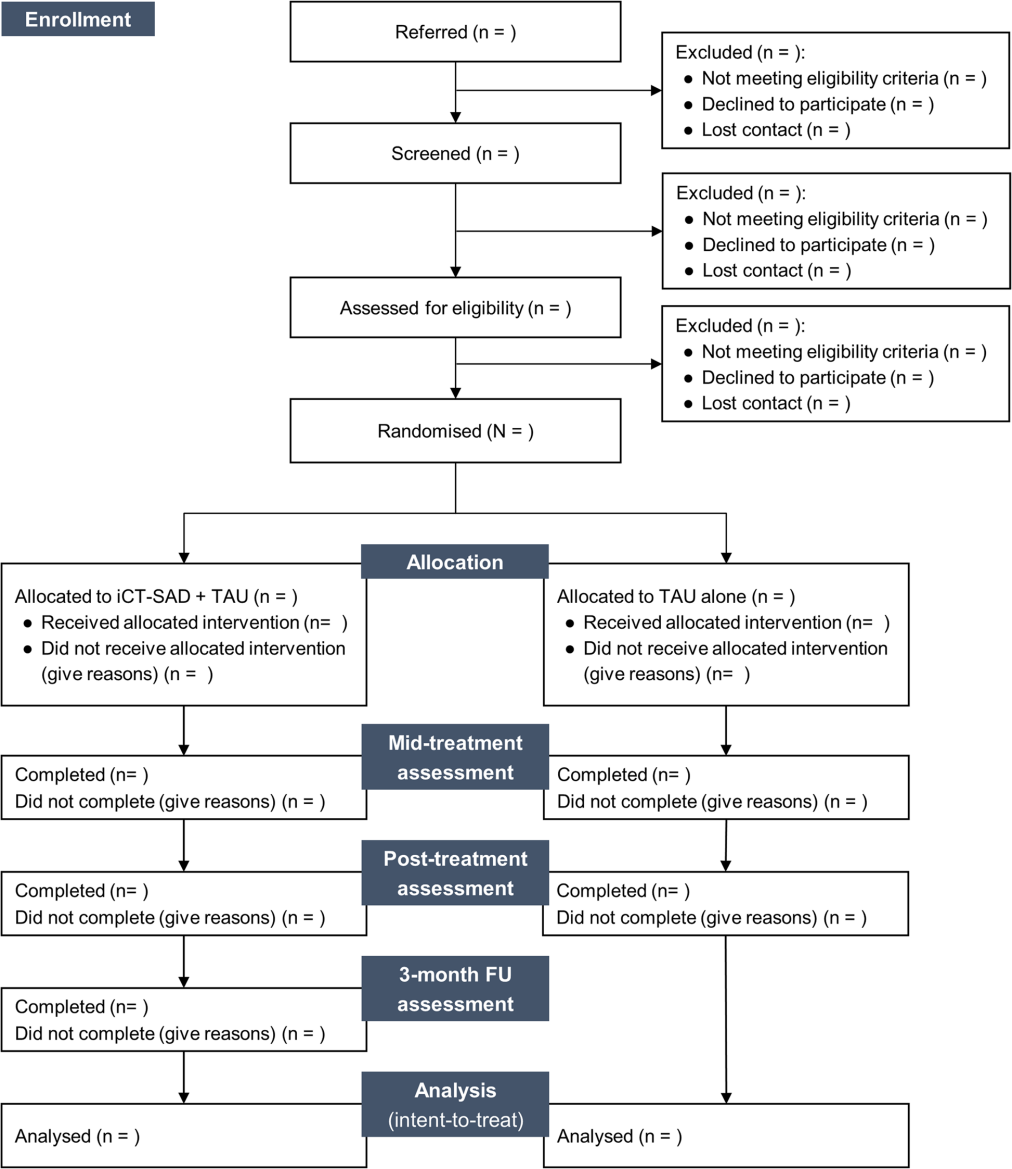
Flow diagram of the trial. iCT-SAD internet-delivered version of cognitive therapy for social anxiety disorder, TAU treatment as usual, FU follow-up

The trial office will contact the participant to inform them whether they are eligible to take part, and if so, to confirm that they are still willing to participate and proceed with randomisation. If they agree to proceed, the assessor will inform the trial office for randomisation. The assessor will inform the participant of the allocation. A trial therapist is then assigned and appointments are arranged. For an overview of the trial procedure, see Table 1 (time schedule) and Fig. 1 (flow diagram).

**Table 1:** Schedule of enrolment, treatments, and assessments

PHASE		Screening and baseline assessment	Allocation	Post-allocation		
				Treatment phase		Follow-up phase
TIMEPOINT		Baseline (week 0)		Mid-treatment (week 8)	Post-treatment (week 15)	3-month FU (week 27)
ENROLMENT	Eligibility screen	X				
	Informed consent	X				
	Demographics	X				
	Allocation		X			
INTERVENTION	iCT-SAD + TAU			➡	➡	➡
	TAU			➡	➡	
ASSESSMENTS	Questionnaires	X		X	X	X
	ADIS-5 SAD	X			X	X
	Adverse events			X	X	X
	Current medication	X		X	X	X
	Acceptability and feedback					X

*FU* follow-up, *iCT-SAD* internet-delivered version of cognitive therapy for social anxiety disorder, *TAU* treatment as usual, *ADIS-5* Anxiety and Related Disorders Interview Schedule for DSM-5, *SAD* social anxiety disorder

### Sample size {14}

Sample size was calculated a priori for the between-group comparison of LSAS scores from baseline to post-treatment. The controlled effect size (Cohen’s *d*) observed in a previous randomised controlled trial of iCT-SAD compared to a waitlist control group with continuing primary care in the UK [8] was 2.2, which equates to a mean difference of 44 LSAS points. However, as the present trial uses a different population and cultural setting and is the first randomised controlled trial of iCT-SAD translated into another language, we used a conservative estimated mean difference of half this value (22 points). This calculation (using *β* = 0.9, *α* = 0.05, and a LSAS standard deviation of 19) indicated a required minimum sample of 16 participants per arm. Given the planned analyses of secondary measures and allowing for attrition (up to 20% dropout), we need 30 participants per group, making a total sample size of 60 to ensure the trial is fully powered to answer the research questions.

### Recruitment {15}

Participants will be recruited from psychiatric institutions in Japan, via referrals from their primary psychiatrist. To ensure sufficient participant recruitment, the trial will be promoted via trial therapists’ networks and relevant medical institutions using posters, leaflets, and digital media.

## Assignment of interventions: allocation

### Sequence generation {16a}

Participants will be randomly assigned to either the intervention or control arm at a ratio of 1:1, with the assignments made by the Internet Data and Information Center for Medical Research (INDICE) cloud, provided by the University Hospital Medical Information Network (UMIN). Randomisation will be stratified by baseline LSAS severity (≥ 78 or less). A minimization algorithm will be used to ensure a balance between arms.

### Concealment mechanism {16b}

The random allocation will be carried out by the UMIN INDICE cloud, an internet-based central randomisation system. Allocation concealment will be ensured, as the allocation result for each participant will not be visible to the trial team before they have been assigned to one of the treatment arms.

### Implementation {16c}

Once an eligible participant has agreed to participate, the assessor will inform the trial office. An independent research assistant will access the UMIN INDICE cloud and enter the necessary baseline information. The random allocation to either arm will be centrally generated. The trial office will inform the therapist of the allocation result and the therapist will then contact the participant.

## Assignment of interventions: blinding

### Who will be blinded {17a}

Owing to the nature of psychological interventions, it is not possible to blind participants or therapists to the intervention condition. A qualified independent assessor who conducts ADIS-5 for SAD at the post-treatment point will be blinded to the intervention condition.

### Procedure for unblinding if needed {17b}

The independent assessor of ADIS-5 for SAD will remain blinded throughout the trial. No other blinding is planned so unblinding will not occur.

## Data collection and management

### Plans for assessment and collection of outcomes {18a}

All participants will be given baseline questionnaires through an online survey platform. Settings will be used to alert participants to items they have missed and require a response to prevent missing data. Participants in the intervention arm will complete questionnaires using the iCT-SAD programme. Participants in the control arm will complete questionnaires through an online survey platform, with email reminders from the trial office. Japanese language versions of the below questionnaires will be used.

LSAS (primary outcome) is a 24-item scale with two subscales, fear/anxiety and avoidance of social situations. Each item is rated on a four-item Likert scale ranging from 0 (no fear/never avoidance) to 3 (high fear/usually avoidance), with lower scores indicating a better outcome. The Japanese version of LSAS has good internal consistency and construct validity [28, 29].

SPWSS is a 6-item scale of self-focused attention, anticipatory processing, post-event processing, and other variables, with each item rated on a scale from 0 to 8 [20].

SCQ is a 22-item scale that assesses the most common negative automatic thoughts related to SAD [20]. Each thought is rated in terms of how often it occurred in the last week and how much it is believed. The mean scores for frequency (mean ranges from 1 to 5) and for belief (mean ranges from 0 to 100) are computed separately, with lower scores indicating a better outcome.

PHQ-9 is a validated scale for assessing depressive symptoms [22, 23]. This scale contains 9 questions answered on a 4-point Likert scale, with numbers from 0 (not at all) to 3 (nearly every day). A higher score reflects more severe symptoms.

GAD-7 is a validated questionnaire commonly used to assess generalised anxiety symptoms and their severity [24, 30]. This scale consists of 7 questions answered on a 4-item Likert scale, with numbers from 0 (not at all) to 3 (nearly every day). A higher score reflects more severe symptoms.

WSAS is a five-item scale that measures perceived functional impairment across five domains: work, home management, social leisure activities, private leisure activities, and relationships with others [26]. Each item is rated on a 9-point severity scale ranging from 0 (not at all) to 8 (very severely), based on the degree to which the issue impedes one's ability to perform an activity. The Japanese version of the WSAS has good internal consistency and construct validity [31].

SBQ is a 29-item scale that assesses the extent to which patients engage in a range of common social anxiety related safety behaviours [20]. Each of the 29 behaviours are rated on a 4-point scale ranging from 0 (Never) to 3 (Always), with lower scores indicating a better outcome.

SAQ is a 50-item scale that assesses a range of common beliefs about oneself and social interactions that are thought to make individuals vulnerable to SAD [20]. The beliefs fall into three broad categories: ‘Excessively high standards for social performance’, ‘Conditional Beliefs’ and ‘Unconditional Beliefs’. Each item is rated on a 7-item Likert scale ranging from 1 (Totally disagree) to 7 (Totally agree), with lower scores indicating a good outcome.

Social participation and social satisfaction are measured with Alden and Taylor’s scales [21]. Social participation has 13 items that ask about the frequency of participation in social interactions, events, and conversations in the last month. Each item is rated from 1 (not at all) to 7 (frequently). Social satisfaction has five items that ask about satisfaction with relationships with colleagues, friends, and partners in the last month. Each item is rated from 1 (not satisfied at all) to 7 (very satisfied).

### Plans to promote participant retention and complete follow-up {18b}

Participants will be in regular contact with their primary psychiatrist and/or iCT-SAD therapist, who will provide support and encouragement, and are available to discuss any concerns participants may have about treatment or trial participation. Trial information and initial assessment procedures will clarify the timescale of treatment and explain the clinical importance of receiving a full ‘dose’ of iCT-SAD. Any participants who withdraw early from either arm will be invited to complete follow-up questionnaires at the relevant remaining principal time points.

### Data management {19}

Most of the data gathered online and saved to a database will be coded automatically. Metadata about participants’ activity on the iCT-SAD programme (e.g. time spent on different parts of the programme) will be automatically recorded. As most of the data will be collected automatically via secure online platforms specifically designed for research purposes, high data quality will generally be guaranteed. Only a small fraction of data (e.g. baseline demographic data, diagnostic assessment by an independent assessor) will be entered and uploaded to a secure server by the assessor or the trial office. The trial office will regularly check the overall data quality and completeness.

### Confidentiality {27}

Personal information of potential and enrolled participants will be collected and stored on a secure server available only to the trial office. Participant’s name, telephone number, and e-mail address will be informed only to the trial office and the trial therapist who will be assigned to them. Upon enrolment, each participant will be assigned a unique participant identification number for use in the data files created for the analysis. Data are kept in a secured storage and only authorised researchers or staff at the trial office are granted access by the principal investigator.

The iCT-SAD programme has numerous security features representing current best practice and complies with high data security standards. It employs secure client–server communication, full encryption of the server database, enforcement of strong passwords, two-factor authentication, and hosting on a tier 4 hosting server. External access to the database using the Secure Shell Protocol is prohibited. The system has been subjected to industry-standard penetration testing. Online data are secured by encryption to prevent access from outside parties. Access to the server data by the software company hosting the online therapy programmes (Whiskered Wizard) is protected by non-disclosure agreements and the Data Protection Act.

### Plans for collection, laboratory evaluation, and storage of biological specimens for genetic or molecular analysis in this trial/future use {33}

Not applicable. See the above item 26b; there will be no biological specimens collected.

## Statistical methods

### Statistical methods for primary and secondary outcomes {20a}

A separate statistical analysis plan (SAP) will be developed and approved by the trial team and an independent statistician before the database is locked. A detailed SAP will be uploaded to the trial registry (ISRCTN82859645). A brief overview of these analyses is given below.

Statistical analysis and reporting of this trial will be conducted in accordance with CONSORT guidelines, with the primary analyses based on the intent-to-treat principle without imputing missing observations. All statistical tests will be two-sided, and the level of significance will be set to 0.05. For the baseline variables, summary statistics are constructed employing frequencies and proportions for categorical variables and mean and standard deviation for continuous variables.

Linear mixed effect models will be used to analyse the primary and continuous secondary outcome measures. Time (mid, post), treatment arm (TAU + iCT-SAD, TAU alone), and the time-by-arm interaction will be specified as categorical fixed factors, with the stratification variable (baseline LSAS) as a fixed covariate and participant as a random effect. For the analysis of secondary outcome measures, the baseline score of the analysed measure is also included as a fixed covariate.

Standardised between-group effect sizes will be calculated by dividing the adjusted group difference by the baseline standard deviation of the whole sample. Standardised within-group effect sizes will be calculated from separate models that use the baseline score as a timepoint rather than a covariate.

Categorical secondary outcome measures (loss of diagnosis, response to treatment, and categorical acceptability outcomes) will be analysed using Fisher’s exact test. All other results will be reported descriptively.

Benchmarking will be performed through descriptive comparisons of the present results in relation to previous studies of iCT-SAD in the UK and Hong Kong [8, 9, 11, 12].

Baseline clinical and demographic characteristics will be examined as moderators of treatment outcomes by running linear mixed effect models (iCT-SAD + TAU only) with each candidate moderator added.

Mediation models will examine candidate mediators of the relationship between randomisation (treatment arm) and post-treatment LSAS scores following the procedure described by Freeman et al. [32]. The candidate mediators will be negative social cognitions (SCQ), safety behaviours (SBQ), self-focused attention (the general self-focused attention item of the SPWSS), rumination (the rumination item of the SPWSS), and depressed mood (PHQ). Mid-timepoint scores are used as the mediator, and post-treatment LSAS scores as the outcome. All models include baseline LSAS and baseline mediator scores as covariates.

### Interim analyses {21b}

No interim analyses are planned.

### Methods for additional analyses (e.g. subgroup analyses) {20b}

No additional analyses are planned.

### Methods in analysis to handle protocol non-adherence and any statistical methods to handle missing data {20c}

Primary analyses will be performed based on the intent-to-treat principle. Data completeness will be evaluated and reported at each timepoint in each arm. Linear mixed effect models implicitly account for data missing at random; therefore, multiple imputation of missing observations is not planned.

### Plans to give access to the full protocol, participant level-data, and statistical code {31c}

The trial protocol was prospectively registered in ISRCTN and UMIN-CTR. After the data have been used for all trial purposes, an anonymised version of the dataset will be deposited in an online repository for permanent archiving to use in future studies or meta-analyses.

## Oversight and monitoring

### Composition of the coordinating centre and trial steering committee {5d}

This trial does not have a coordinating centre or trial steering committee. The principal investigator (NY) is responsible for the overall aspects of the trial. The trial team meets every week to oversee the progress of the trial and the completion of tasks and to review necessary changes to the protocol to facilitate the smooth running of the trial. Any major changes to the trial protocol will be reviewed and verified by the Research Ethics Committee.

### Composition of the data monitoring committee, its role, and reporting structure {21a}

Due to the low-risk nature of the intervention, the short duration of the trial, and the fact that interim analyses may not provide necessarily informative results given the nature of the trial, the establishment of a data monitoring committee is not considered necessary. All outcome measures are coded directly into a web programme that cannot be adjusted with all actions being logged. Data will be monitored for completeness and consistency by the trial team. The trial team and statistician will have full access to the final trial dataset.

### Adverse event reporting and harms {22}

All adverse events (AEs) and severe adverse events (SAEs), which may or may not be related to treatment, will be logged throughout the trial. Based on the guidance of Rozental et al. [33], these include the following:

- Worsening of SAD symptoms
- Negative events probably emerging from treatment perceived as adverse by the patient
- Emergence of new psychological symptoms unrelated to those targeted in treatment (e.g. new occurrence of insomnia, panic attacks, etc.).
- External negative events in participant’s life (e.g. bereavement, other stressors)
- Other events experienced as negative by the patient during treatment, which may or may not be related to treatment (e.g. issues related to treatment content, increased anxiety during experiments, technical issues).

SAEs are defined as negative events that occur during treatment that require some form of urgent alternative treatment (e.g. substance misuse, deliberate self-harm, suicidal ideation or attempts).

Trial therapists will identify and record their participants’ AE/SAEs when providing iCT-SAD treatment. Participants in both arms will also be asked to report any negative effects experienced during the trial, via a checklist included in the mid-, post-treatment, and 3-month FU assessment packs. AEs will be reviewed on a regular basis by the trial team, including in clinical supervision. SAEs will be brought to the project lead’s attention immediately to consider what actions may be appropriate. This would likely include contact with the participant’s primary psychiatrist*.*

### Frequency and plans for auditing trial conduct {23}

No audit is planned for trial conduct. The principal investigator is obliged to and will inform the Research Ethics Committee in case of any major deviations from the trial design approved by the Committee.

### Plans for communicating important protocol amendments to relevant parties (e.g. trial participants, ethical committees) {25}

All protocol amendments will follow the Research Ethics Committee revision process and will be approved prior to their implementation. Where necessary, the trial team members will be informed via email or in clinical supervision. Deviations from the published protocol will be documented in the trial registration on ISRCTN and UMIN-CTR.

#### Dissemination plans {31a}

The findings of this trial will be disseminated to academic and professional audiences via publications in peer-reviewed journals and presentations at academic conferences, and to the broader public via public talks and media/press releases. All findings from the trial, whether negative or positive, will be reported.

## Discussion

Investigating the efficacy of internet interventions in different cultural contexts is an important step in determining whether the potential global reach of such treatments can be achieved. This trial provides a valuable examination of iCT-SAD combined with usual care, which is a good reflection of how the treatment might subsequently be provided in routine settings. It offers a further contribution to the small but growing literature on the international dissemination of internet interventions and to our understanding of treating social anxiety in Japan. A limitation of this trial is that an active comparator arm will not be employed, meaning the specific effects of iCT-SAD relative to other internet interventions cannot be revealed.

## Trial status

Protocol version 1.0, 2023-October-18. The study is planned to begin in November 2023 and is estimated to be completed in December 2025. Recruitment is planned to begin in January 2024 and is estimated to be completed in May 2025.

## Data Availability

A de-identified individual-level dataset will be made available through a publicly accessible online repository after the primary findings of this trial are published.

## Abbreviations

ADIS-5: Anxiety and Related Disorders Interview Schedule for DSM-5
AE: Adverse events
CT: Cognitive therapy
CONSORT: Consolidated Standards of Reporting Trials
DSM-5: Diagnostic and Statistical Manual of Mental Disorders, Fifth Edition
FU: Follow-up
GAD-7: Generalised Anxiety Disorder Questionnaire 7-item
iCT-SAD: Internet-delivered cognitive therapy for social anxiety disorder
INDICE: Internet Data and Information Center for Medical Research
ISRCTN: International Standard Randomized Controlled Trials
LSAS: Liebowitz Social Anxiety Scale
NICE: National Institute for Health and Care Excellence
PHQ-9: Patient Health Questionnaire 9-item
SAD: Social anxiety disorder
SAE: Severe adverse event
SAQ: Social Attitudes Questionnaire
SBQ: Social Behaviour Questionnaire
SCID-5: Structured Clinical Interview for DSM-5 Disorders
SCQ: Social Cognitions Questionnaire
SPWSS: Social Phobia Weekly Summary Scale
TAU: Treatment as usual
UMIN: University Hospital Medical Information Network
UMIN-CTR: UMIN Clinical Trials Registry
WSAS: Work and Social Adjustment Scale
